# Organosilicon Self-Assembled Surface Nanolayers on Zinc—Formation and Their Influence on the Electrochemical and Corrosion Zinc Ongoing

**DOI:** 10.3390/ma16176045

**Published:** 2023-09-02

**Authors:** Maxim Petrunin, Liudmila Maksaeva, Tatyana Yurasova

**Affiliations:** A.N. Frumkin Institute of Physical Chemistry and Electrochemistry, Russian Academy of Sciences, Moscow 119071, Russia; lmaksaeva@mail.ru (L.M.); tatal111@yandex.ru (T.Y.)

**Keywords:** adsorption, organosilane, zinc, self-assembled siloxane layers, corrosion

## Abstract

The adsorption of vinyltrimethoxysilane (VS) on the surface of sputtered (by thermal spraying in vacuum) zinc has been investigated. The adsorption isotherms of VC on zinc from an aqueous solution were obtain. In order to determine the adsorption characteristics of VS molecules and to calculate the heats of adsorption, the obtained adsorption isotherms were mathematically processed in terms of the well-known adsorption approaches (approximations, adsorption isotherms). It has been established that this organosilane was chemisorbed on the surface of freshly deposited zinc after 60 min. After the sample was immersed in the solution, a self-organized organosilicon layer was formed on the metal surface. The application of Fourier transform infrared spectroscopy, atomic-force microscopy, and scanning electron microscopy allowed to us study in detail the interactions between VS molecules and the metal surface and to determine the structural features of the formed surface films. The mechanism of formation of self-assembled surface layers on zinc has been proposed. Electrochemical and corrosion research methods have been used to investigate the anticorrosion characteristics of organosilicon films on zinc. High stability of surface organosilicone layers with respect to the corrosive components of electrolyte action was shown by an infrared spectroscopy study carried out after corrosion tests

## 1. Introduction

Zinc is a non-ferrous metal widely used in industry [1,2]. Zinc alloys are used as sacrificial anodes in electrochemical (cathodic) corrosion protection systems for ships and/or underground and subsea metal structures from corrosion [1]. However, zinc is most widely used for coating steel products (for example, sheets) to obtain galvanized steel. For example, steel galvanization and zinc-rich coatings for c steel products operated in the atmosphere and in water account for approximately 45% of the production volume of zinc in the world [2,3,4].

The use of zinc in numerous diverse areas is mainly due to its high corrosion resistance under natural conditions [2,3]. However, despite this, zinc can undergo both uniform and local corrosion [3,5,6] that can damage the integrity of a zinc coating. Such damage jeopardizes the reliability of a steel structure since the corrosion rate is higher in areas with damaged coatings than in coated areas. This can result in perforation of the structure wall and its premature failure. To preserve the appearance and prevent the corrosion of zinc products and zinc coatings, they are often passivated, i.e., subjected to additional chemical treatment by short-term immersion in passivating solutions. Of these, solutions based on chromic acid, or its salts are the most common. Chromate treatment of zinc at the same time simultaneously promotes inhibition corrosion of the metal from corrosion and improves adhesion of the polymer or paint coating. However, hexavalent chromium, which is the main component of the passivating chromate formulations used in the treatment, is a mutagenic and carcinogenic compound, the use of which is undesirable for environmental reasons. Nowadays, it is banned in some developed countries, and in the near future, a complete ban of technologies involving the use of hexavalent chromium compounds can be expected [7,8,9].

In recent decades, an environmentally friendly alternative to metal chromate treatment has been actively searched for [10,11,12,13]. However, despite the advances in the search for chromate-free methods of metal treatment, no universal method that could successfully compete with chromate treatment for a wide range of metals and operating conditions has been suggested to date.

Alkoxysilanes (with the chemical structure: RnSi(OR’)4-n) are environmentally friendly compounds [14,15] that are able to adsorb on inorganic substrates surfaces (for example, metals), forming surface self-assembled siloxane nanolayers bonded to the surface through strong and hydrolytically resistant bonds of the type Me-O-Si [16,17,18,19,20,21,22,23,24].

Such layers contain reactive groups (R), which can react with the functional groups of many polymeric coatings. This feature has been successfully used for many years to increase the strength and stability of inorganic substrate-polymer adhesive joints. For example, in composite materials [17,20]. Moreover, organosilicon nanolayers on the metal surfaces are able to improve its corrosion resistance [16,18,19,20,21,22,23,24,25,26,27,28]. Vinyltrimethoxysilane CH_2_=CHSi(OC_2_H_5_)_3_ (VS) is an inexpensive, environmentally friendly, and commercially available reagent. Our previous works [16,19,23,24] present the results of studying the adsorption of VS and the formation of siloxane nanolayers on the surfaces of copper, aluminum, iron, and carbon steel.

The objective of this work was the investigation of vinyltrimethoxysilane adsorption on the zinc surface, and the study of the features of the formation of surface vinylsiloxane layers, as well as the evaluation of these layers’ influence on the zinc electrochemical and corrosion properties.

## 2. Materials and Methods

We used vinyltrimethoxysilane CH_2_=CH-Si(OCH_3_)_3_ (VS) of ‘special purity’ grade, bulk high purity zinc (99.997%Zn), and zinc foil of TsV0 grade, 0.9 mm thick, with a zinc content of 99.995%.

Zinc coatings on metals are obtained by various methods, for example, plasma-electrochemical, galvanic, and physical methods for obtaining (deposition) coatings. Physical methods of vacuum thermal sputtering seem to be the most suitable for model physicochemical studies due to the high purity of the resulting zinc films. In addition, varying vacuum thermal sputtering mode makes it possible to obtain metal coatings with controlled thickness, ordered structure, and unique functional properties [27,29,30].

In this regard, to ensure the reliability and reproducibility of adsorption studies in this work, the VS adsorption has been investigated on the surface of a layer of zinc thermally sputtered in vacuum in the following way: A weighed zinc sample has been heated to the evaporation temperature in a tungsten evaporator by passing an electric current with current amperage equal to 16 A, in a vacuum 10–6 mm Hgt. (vacuum plant VUP 4).

The following substrates were used for the deposition of zinc layers:

(a) gold-coated quartz resonator with AT-cut, main resonance frequency 10 MHz, brand QC-10-AuBU (manufactured by Elchema, Potsdam NY 13676 USA):
(b) silicate glass (rectangular samples 1.5 × 3.0 cm, 2 mm thick), the surface of which has been purified by ethanol before metal deposition, then rinsed with bidistillited water and dried in air at room temperature. VS adsorbed onto zinc from an aqueous solution. If further in the text the term “solvent” is mentioned, then it means water, and if “solution” is mentioned, then it means “aqueous solution”.

Adsorbed VS weight has been determined by piezoquartz nanobalance technique [31,32] by measuring the change in the oscillation of the quartz resonator frequency (Δ*f*, which is determined by the mass deposited on the quartz surface and is calculated by Formula (1) [32]

(1) $$\Delta m=-(N\rho S\Delta f/f_{0}^{2})$$

where *f_0_* is the fundamental frequency of the quartz resonator (kHz)). Δ*f*—measured frequency shift (kHz). Δ*m* is the value of the mass deposited on the piezoquartz resonator (g); *N* is the frequency quartz constant, which depends on the of the ridged quartz plate orientation with respect to the main crystallographic axes of the quartz crystal (for example:, *N* = 1670 kHz mm in the case of AT-cut crystals); *ρ* is quartz density, equal to 2.65 g/cm^3^; and *S* is quartz working area, equal to 0.72 cm^2^

An electrochemical piezoquartz nanobalance device Elchema EQCN 700 (Elchema Inc. Potsdam NY 13676 USA) was used. The sputtered metal mass has been recalculated to layer thickness, supposing a uniform distribution of sputtered zinc on the surface of a substrate. The thickness of the resulting layer was 1.1 ± 0.1 μm. After the deposition finalization, the working space was immersed in vacuum for 30 min, after that, the air was launched into the working cell. The samples with sputtered zinc were removed from the cell and placed in a desiccator with dehydrated CaCl_2_. Adsorption studies were carried out 15 h after the metal film preparation.

The adsorption of vinytrimethoxysilane has been studied by “in situ” quartz nanobalance in solution by measuring the change in the frequency of the quartz resonator after adding organosilane to the solution. Upon reaching a constant frequency value and the cessation of oscillations, samples with adsorbed layer of VS were kept in the solvent to remove the reversibly adsorbed organosilane molecules. The mass of adsorbed silane was calculated by Formula (1).

The zinc surface morphology has been studied “ex-situ” (in air) using an atomic force microscope brand Solver-Pro (manufactured by NT-MDT, Zelenograd, Russian Federation) using contact regime. The data were software processed to obtain images and to fix the roughness of surface values by applying the NOVA Solver Pro software package (NT-MDT, Zelenograd, Russia). Images have been obtained using the WSxM software package [33].

To study the electrochemical behavior of zinc, anodic polarization potentiodynamic curves were recorded in a 0.01 M solution of NaCl. The studies were carried out using a cylindrical zinc electrode (high pure zinc, 99.997% Zn). Electrochemical investigations have been carried out in a standard electrochemical setup (three-electrode setup) applying a potentiostat brand IPC-Pro MF. There was a platinum auxiliary electrode (1.2 cm^2^) and a chloride-silver reference electrode.

In the first 60 s after the sample was immersed in the solution, the value of corrosion potential (*E_cor_*) was recorded. Then, the anodic potentiodynamic polarization curves (with a potential sweep rate equal to 0.1 mV/s) were recorded by shifting the electrode potential from the stationary value *E_cor_* in the positive direction to potential values appropriated to the localized (pitting) anodic dissolution with a stable rate of pitting development. The values of critical pitting potential (*E_pit_*), i.e., the potential above which pitting dissolution of the metal occurs and stable pitting appears on a surface [34], were found from the break in the anodic polarization curve as the potential magnitude after which a sharp increase in anodic current occurs [34,35]. As a criterion for the effectiveness of braking localized anodic dissolution after the modification of the metal surface, the shift of the electrode pitting potential (*E_pi_*_t_) [36] towards more positive values was chosen:

(2) $$\Delta E=E_{pit}-E_{pit}^{mod}$$

*E_pit_*—potential of pitting formation of an unmodified metal, *E_pit_^mod^*—potential of the pitting formation of a metal modified with vinylsilane solution.

In addition, the influence of obtained organosilicon surface layers on a metal anodic dissolution was evaluated by measurement of the anodic current value at a potential more positive than the pitting potential: The lower the current density, the more efficiently the surface layer inhibits the zinc anodic dissolution.

Corrosion tests were performed using foil samples 2 × 3 cm in size, bulk samples 3 mm thick and 3 × 5 cm in size, QC-10-AuBU quartz resonators (Elchema, P.O.Box 5067 Potsdam, New York, NY 13676, USA), and glass plates 15 × 30 × 2 mm in size with thermally applied zinc layers.

For accelerated corrosion testing, a heat-moisture test chamber (brand MNK-408CL (manufactured by Terchy, Taiwan) at RH 95%, t = 60 °C) and a sodium chloride solution with a range of concentrations from 0.001 to 0.1 M was applied. A cylindrical glass cuvette with a sleek, even, and light transparent bottom was used for the experiments in solutions. A sample was installed at the bottom of the cuvette with the studied surface facing down and a gap between the surface of a metal and the glass cuvette bottom was created by using a glass limiter. The metal corrosion potential changing and “in situ” reflectograms of diffuse reflection [37] of the zinc surface were simultaneously recorded using potentiostate IPC Pro and a standard Epson Perfection 3200 Photo computer scanner. The optical resolution of the scanner was 3200 dpi.

The potential was measured with regard to the chlorine-silver chloride reference electrode by means of a digital high-resistance multimeter brand APPA 109N (manufactured by АРРА Technology Corporation" Taipei City, 114 Taiwan). A magnitude of the potential was converted to the normal hydrogen scale. All the potentials presented here are given with respect to the standard hydrogen electrode (SHE).

The metal corrosion rate was estimated by the following methods: in situ piezoquartz nanoweighing, resistometry [38], gravimetry (analytical balance), and scanner reflectometry [35].

In the piezoquartz weighing method, the corrosion rate was estimated from the changes in the mass of the sample, which was calculated using Equation (1).

In the resistometric assessment of corrosion, the electrical resistance of the metal film during corrosion was controlled, and from the change of metal resistance, the change in the layer thickness Δ*d* (mm) or the average corrosion rate (*K*, mm/y) was calculated by means of Equations (3) [36] or (4) [39], respectively:

(3) $$\Delta d=d_{0}-d_{\tau }=(\Delta l/h)(1/R_{0}-1/R_{t})$$

(4) $$K=365\;d_{0}(1-R_{0}/R_{\tau })\tau$$

where *d_0_* is the initial metal layer thickness of (mm), *τ* is the time of corrosion test (days), *R_o_* is the initial electric resistance of the sample, *R_τ_* is the resistance of the sample at the testing time *τ*.

The electrical resistance of samples was measured during corrosion tests with an accuracy of 0.01 Ω during corrosion tests in continuous mode with a polling interval of 10 min using an APPA109N multimeter (АРРА Technology Corporation Taipei City, 114 Taiwan). The multimeter provides automatic fixation and storage of measured values. For gravimetric assessment of corrosion, samples were weighed before and after testing on an electronic analytical balance brand AF-R220CE (Shinko Denshi Ltd., Tokyo 173-0004 (Vibra), Japan). Then, the change in sample thickness was calculated from the mass loss. The corrosion rate was calculated (mm per year), knowing the test time. Before weighing specimens after corrosion testing, the corrosion products had been removed from metal surfaces by standard methods [40,41]. In parallel, three samples were weighed, and the average mass loss was determined and then corrosion rate was calculated.

The localized changes in the reflectivity of samples obtained by scanner reflectometry were visualized by digital image processing that is usually applied to study surface morphology in detail [42,43].

The coverage of the surface by corrosion products was calculated using our own software for image processing written in Ruby 1.9.0 programming language and the RMagick 2.12.0 program (ImageMagick 6.5.6-8).

The changing situation of zinc surface was characterized by “in situ” quartz nanobalance and “ex situ” methods of surface examination, namely, optical and atomic force (AFM) microscopy (AFM), and infra-red spectroscopy (with Fourier transform signal processing) (FT-IR). Optical microscopy was performed using a Carton SPZT50 microscope, Carton Optical (siam) Co.,Ltd. Pathumthani, 12120 Thailand (200× magnification). An amplified image of the surface area was recorded by means of a digital CMOS video camera brand Amoyca AC-300, Cangshan, Fuzhou, Fujian, China. The camera resolution was 2048 × 1536 pixels. The anti-corrosion efficiency of siloxane layers was obtained by the value of the corrosion inhibition coefficient [44,45]:

(5) $$\gamma =K/K_{inh/\mathrm{mod}}$$

*K*—the rate of corrosion of unmodified metal, and *K_inh/mod_*—the corrosion rate of the metal after modification.

The larger the γ value, the more efficient the corrosion inhibition.

IR specular reflection spectra were registered using a Hyperion 2000 IR microscope (36× magnification) coupled with an IFS-66v/s vacuum Fourier transform infrared spectrometer (Bruker, Corporation Camarillo, CA 93012 USA) with a resolution of 2 cm^−1^ in the range of 600–4000 cm^−1^. The spectra were processed using the OPUS software (Bruker, Corporation Camarillo, CA 93012 USA). Correction using the Kramers–Kronig transformation was performed automatically.

## 3. Results

**Figure 1:**
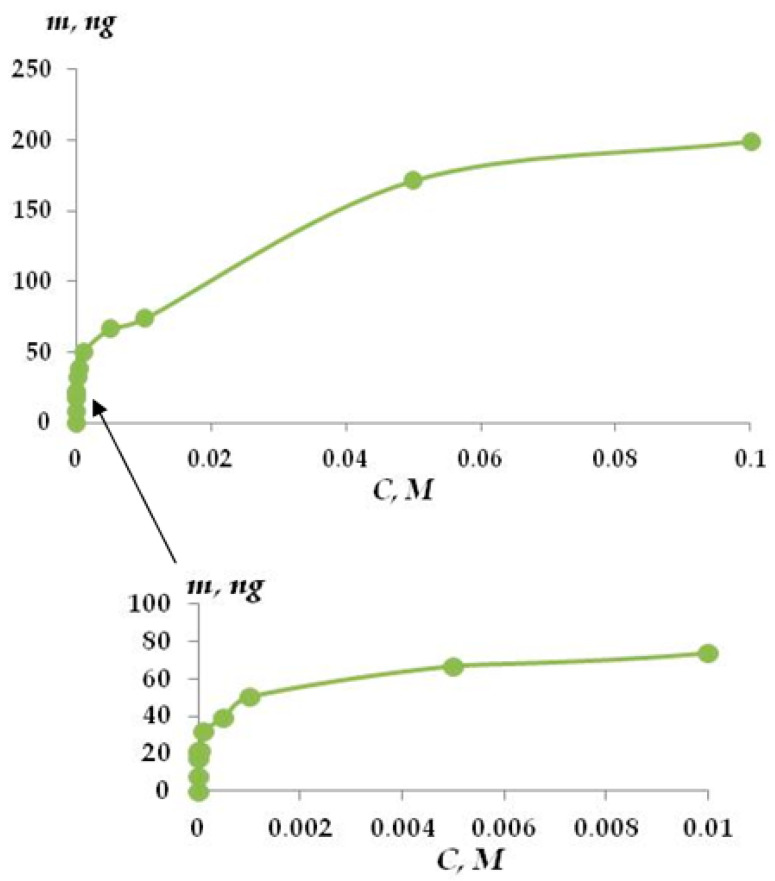
Isotherm adsorption of VS on the zinc. Quartz nanobalance.

When studying the VS adsorption on copper and aluminum surfaces, it was shown [19,22] that the treatment of metal surfaces with aqueous solutions of VS provides the formation of self-assembled nanolayers on the surfaces of the metals. The thickness of these layers depends on VS solution concentration. Vinyltrimethoxysilane adsorption on the zinc was defined by a quartz nanobalance. Isotherm adsorption of VS on the zinc is shown in Figure 1. At low concentrations (up to 1 × 10^−4^ M), pre-monolayer filling of the surface is observed. Monolayer filling is achieved at a concentration of the VS solution equal to 1 × 10^−4^ M. The shape of the isotherm curve at low concentrations corresponds to the Langmuir isotherm. In this case, the mass of adsorbed VS was 20 ng.

**Figure 2:**
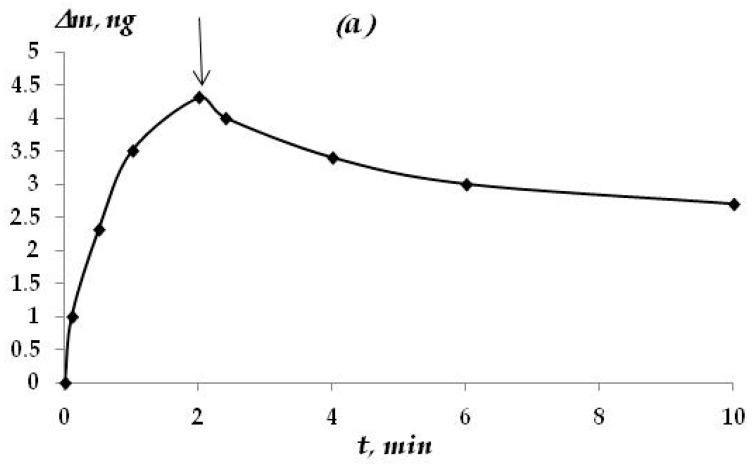

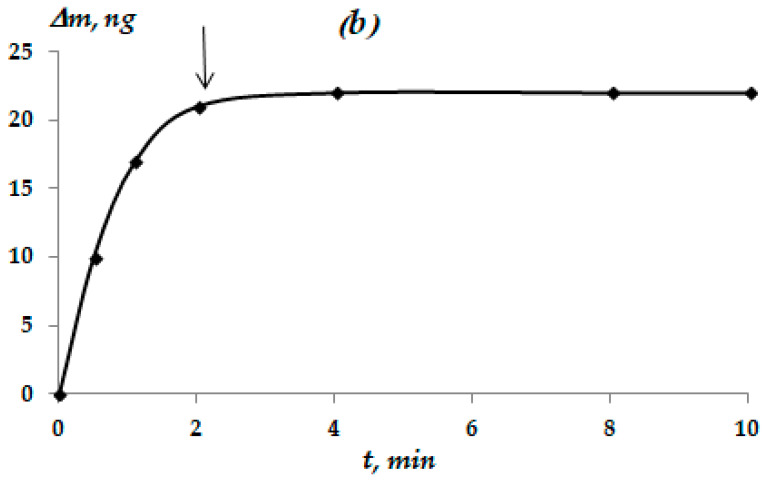
Change in the mass of adsorbed VS in water. VS concentration on the zinc surface: (**a**) 1 × 10^−4^ M; (**b**) 5 × 10^−1^ M. ↓—holding the sample in a pure solvent.

It was found that the solution concentration is less than 5 × 10^−3^ M. The film of adsorbed VS consists of two parts: (1) reversibly sorbed and (2) irreversibly sorbed. Reversibly adsorbed silane molecules are easily desorbed when the specimen has been kept in pure solvent for 10 min (Figure 2a).

Upon reaching the concentration of the solution 1 × 10^−2^ and above, the mass of the sample stopped decreasing during exposure to the solvent (see Figure 2b). This may mean that at such a solution concentration, the reversibly adsorbed amount of silane is negligibly small.

The surface layer, consisting of irreversibly adsorbed molecules, was resistant to the action of water and was not removed from the surface for at least 4 h of exposure to the solvent. The mass of the sample during this time virtually did not change.

**Figure 3:**
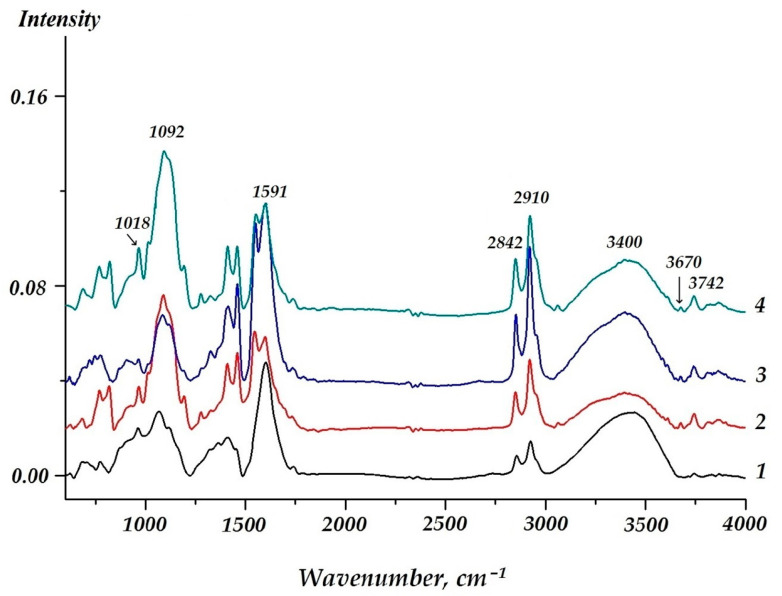
IR spectra. Zinc sprayed, modified VS: (1) 1 × 10^−6^ M; (2) 1 × 10^−4^ M; (3) 0.01 M; (4) 0.1 M.

In order to study the chemical processes that occur during the formation of surface vinylsilane layers formation on zinc, the surface of sputtered metal with an adsorbed vinylsilane layer, as investigated by Fourier transform IR spectroscopy. IR spectra of vinylsilane of various concentrations are shown in Figure 3. The shape of these spectra is the same. The bands near 3450–3500 cm^−l^, at 1591, 550, 1092, and at 2910 cm^−1^, and are most pronounced in the spectra.

**Figure 4:**
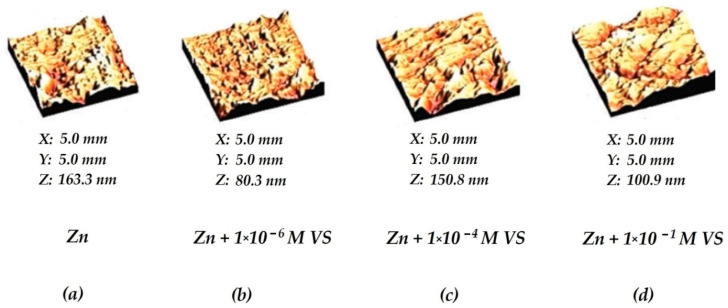
Images of the surface of freshly sputtered zinc after adsorption of VS from aqueous solutions from different concentrations obtained by AFM. (**a**) Original surface ([VS] = 0); (**b**) [VS] = 1 × 10^−6^ M; (**c**) [VS] = 1 × 10^−4^ M; (**d**) [VS] = 1 × 10^−1^ M.

In addition to IR spectroscopy, the parameters of the vinylsiloxane layer formed on the zinc surface were determined using atomic force microscopy (AFM). Figure 4 schematically shows the processed AFM images of surface layers formed on zinc from aqueous solutions, and Table 1 displays the surface roughness of zinc determined from AFM data after modifying the surface of freshly applied zinc with VS solutions.

Figure 4 and Table 1 show that if vinylsilane is adsorbed from low-concentration solutions, the mean surface roughness decreases. A rise in the concentration of the VS s induces a change in the roughness of the metal surface. For example, adsorption of 1 × 10^−6^ M VS reduces the average surface roughness compared to the unmodified surface from 17 nm for the unmodified metal to 7 nm upon modification from a 1 × 10^−6^ M solution of VS in water (Table 1). Increasing the solution concentration to 1 × 10^−4^ M almost doubles the average roughness, and if a 0.1 M VS solution is used, a slight decrease in the roughness value is observed (Table 1).

**Figure 5:**
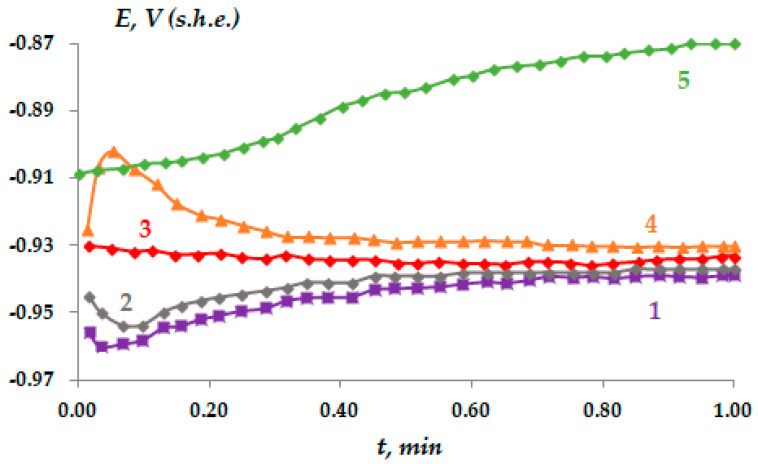
Kinetics of change in the corrosion potential of zinc, 0.1 M NaCl solution, pH 6.7: (1)—Zn without vinylsiloxane layer; (2)—Zn with vinylsiloxane layer, 1 × 10^−^ M VS; (3)—Zn with vinylsiloxane layer, 1 × 10^−4^ M VS; (4)—Zn with vinylsiloxane layer, 1 × 10 ^−−3^ M VS; (5)—Zn with vinylsiloxane layer, 1 × 10^−1^ M VS.

The electrochemical and corrosion ongoing of zinc coated with VS was investigated. The change in the potential of corrosion of zinc in 0.1 M neutral sodium chloride solution is presented in Figure 5. In Figure 5, it can be seen that modification of the zinc surface with a VS solution shifts the corrosion potential of the metal in the positive direction (Figure 5, compare curve 1 and curves 2–5).

**Figure 6:**
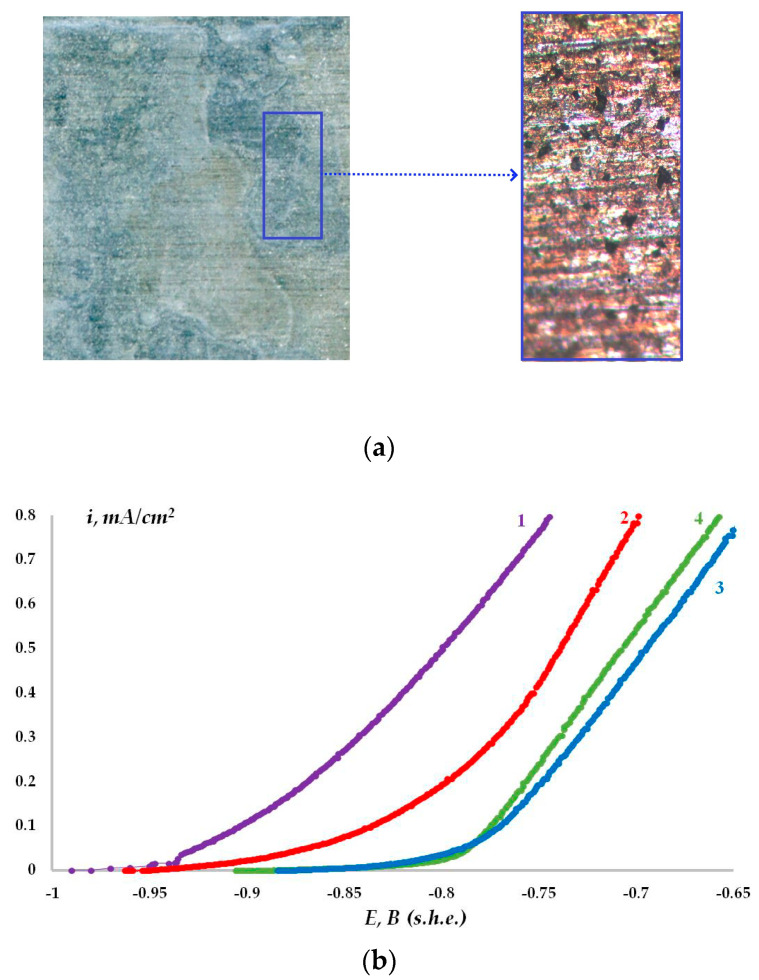

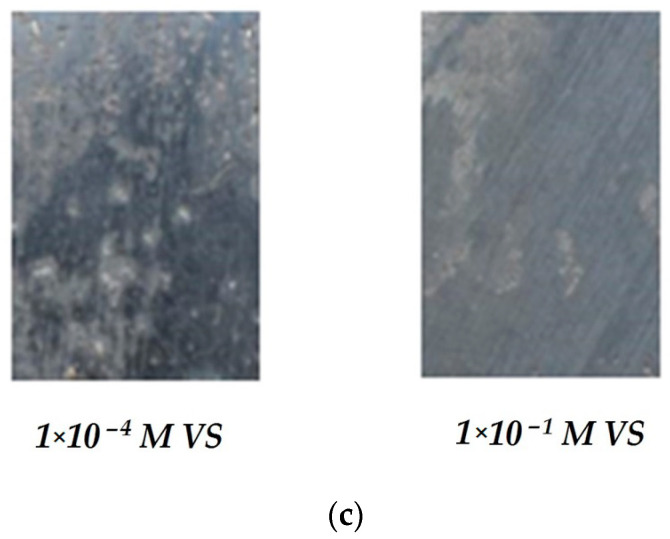
Dissolution of zinc under the action of the anodic potential. (**a**) Appearance of samples of unmodified zinc after pitting dissolution at a potential of −0.75 V (SHE); (**b**) Anodic potentiodynamic polarization curves: (1)—Zn without vinylsiloxane layer: (2)—Zn with vinylsiloxane layer, 1 × 10^−4^ M VS; (3)—Zn with vinylsiloxane layer, 1 × 10^−2^ M VS; (4)—Zn with vinylsiloxane layer, 1 × 10^−1^ M VS; (**c**) Appearance of zinc samples modified with 1 × 10^−4^ M VS solution and 0.1 M VS solution after polarization at E = −0.75 V (SHE) in 0.1 M NaCl working electrolyte. The potential scan rate used to record the potentiodynamic curves is 0.1 mV/s.

In the study on the influence of organosilicon layers on the electrochemical zinc ongoing, the volammetric graphs (anodic curves) of the metal modified with vinylsilane solutions with various concentrations were recorded. Figure 6b displays the corresponding polarization curves.

The polarization curves (Figure 6b) can be used to determine the values of the critical pitting potentials (E_pit_) of zinc modified with vinylsilane solutions. The results are shown in Table 2 from which it can be seen that preliminary modification of the metal surface leads to an increase in the pitting potential.

In addition, to assess the influence of surface layers on the zinc electrochemical ongoing, we used the value of currents of anodic dissolution of the metal at a potential of −0.75 V. The results displayed in Table 3 show that the modification of the surface with vinylsilane provides a reduction of the rate of zinc anodic dissolution (anodic current density).

As can be seen from the anodic curves (Figure 6b, Table 3), the siloxane surface film formed on zinc in a 1 × 10^−4^ M VS solution reduces the current density of anodic dissolution of zinc by almost a factor of two (Figure 6b, compare curves 1 and 2), Table 3: 0.76 and 0.42 mA/cm^2^ for unmodified and modified zinc accordingly. The modifying solution concentration increase to 1 × 10^−3^ M or 1 × 10^−1^ M led to an even greater decrease in the anode current, to 0.22 or 0.19 mA/cm^2^, respectively (Figure 6b, curves 3,4, Table 1 and Table 3), i.e., more than three times compared to unmodified zinc.

**Figure 7:**
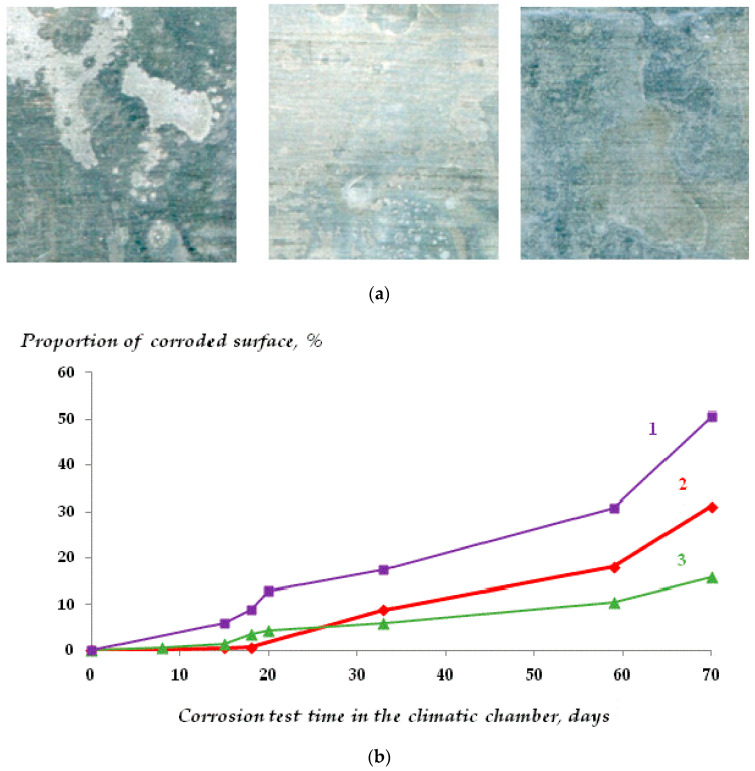

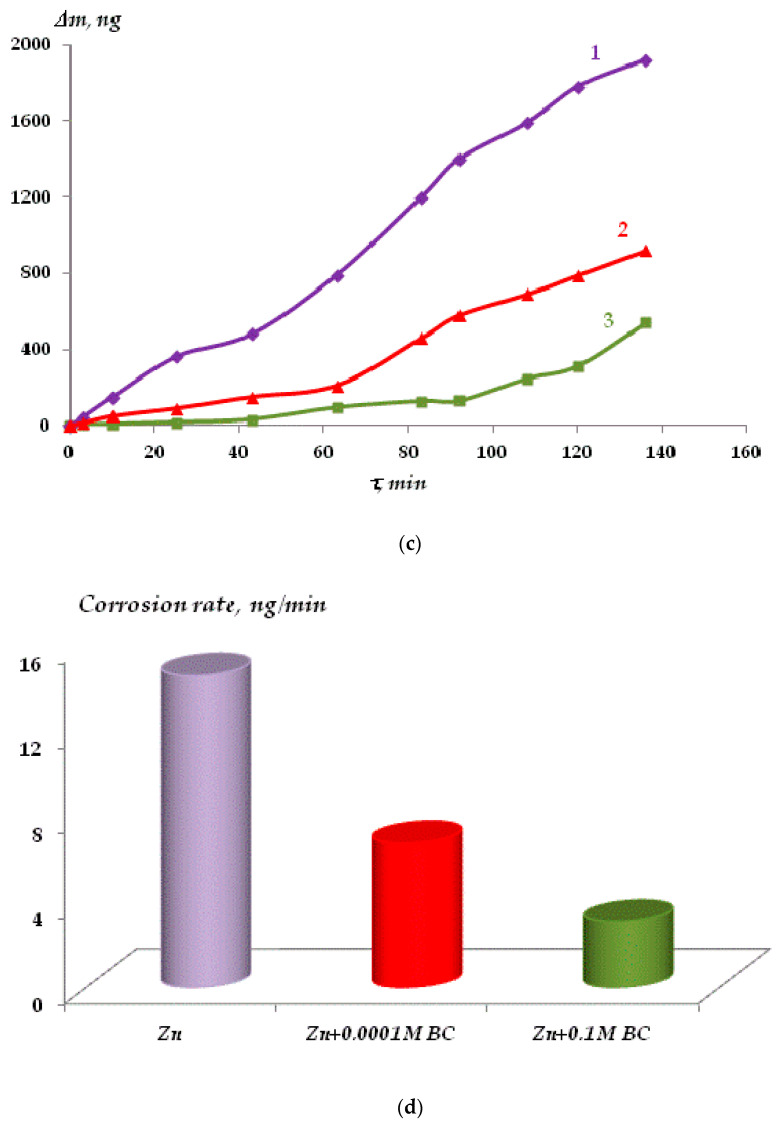

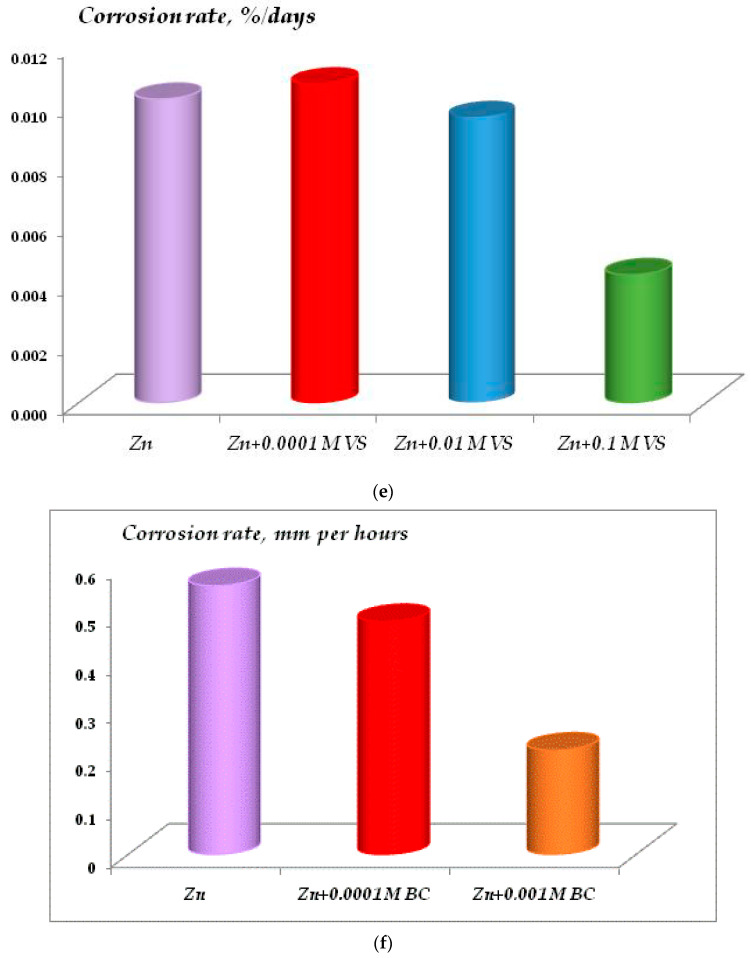
Zinc accelerated corrosion testing, a heat-moisture test chamber, t = 60 °C, RH 95%. (**a**) Appearance of specimens after 112 days of testing; (**b**) kinetics of corrosion of bulk zinc based on the results of corrosion image processing: 1—Zn without a surface nanolayer; 2—Zn modified with 1 × 10^−4^ M solution of VS; 3—Zn modified with 0.1 M solution of VS; (**c**) corrosion rate of freshly sputtered zinc after 112 days of testing. Corrosion rate was estimated by gravimetry, in situ quartz nanoweighing; (**d**) corrosion rate of freshly sputtered zinc after 112 days of testing. Corrosion rate was estimated by processing corrosion images; (**e**) corrosion rate of massive zinc (foil) after 112 days of testing. Corrosion rate was estimated by processing corrosion images; (**f**) Corrosion rate was estimated by resistometry.

The corrosion behavior of zinc was studied. The results of accelerated corrosion tests are shown in Figure 7. Tests were carried out in a climatic chamber. It has been found by using software image processing, determining the degree of filling of the surface with corrosion products (Figure 7b,e) and gravimetrically by quartz nanoweighing (Figure 7c,d) that siloxane layers on the surface reduce the corrosion rate. Moreover, while inhibition of corrosion is insignificant if vinylsilane is applied from diluted solutions ([VS] ≤ 1 × 10^−4^ M), with a modifying solute concentration increase to 1 × 10^−2^ M or 1 × 10^−1^ M, the corrosion rate over 112 days of testing decreases (Figure 7d,e). Resistometric results confirm these data (Figure 7f). It shows that at a modifying solution concentration of 0.1 M, the zinc corrosion rate decreases three times.

**Figure 8:**
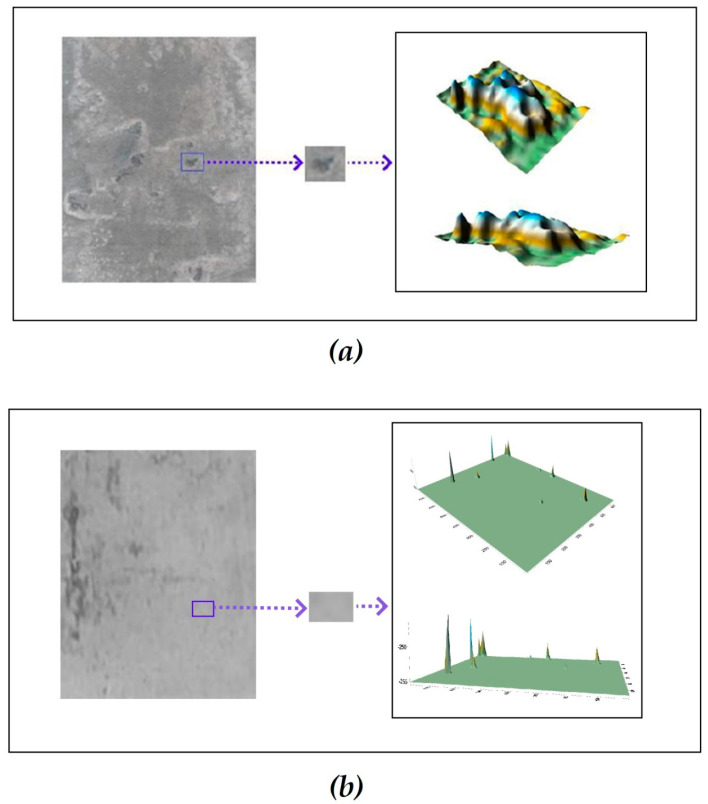
Appearance of zinc samples after corrosion tests in 0.01 M NaCI solution, pH 6.7, duration of 3 days. (**a**) unmodified zinc; (**b**) zinc modified by 1 × 10^−4^ M aqueous VS solution.

It was also shown that pittings up to 100 μm in size were observed on the surface of unmodified zinc in 0.01 M solution of sodium chloride after three days of testing (Figure 8). The presence of one vinylsilane monolayer on the metal surface (modification by 1 × 10^−4^ M VS solution) prevents the formation of large corrosion defects (pittings) only very small defects of 1–2 microns in size were observed on the surface (Figure 8b). When modifying the zinc surface with vinylsilane, uniform dissolution was observed without pits.

**Figure 9:**
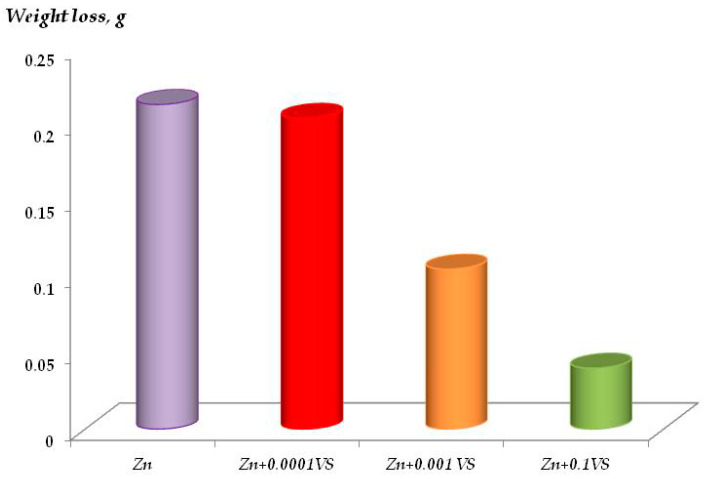
Zinc corrosion in 1 × 10^−2^ M solution of sodium chloride, pH 6.7 after corrosion tests with 2 days duration. Gravimetry method.

It has been established that the degree of inhibition of metal dissolution is determined by the concentration of the modifying solution. For example, if the corrosion images processing results show that the surface films formed during adsorption from 1 × 10^−4^ M, VS solution slightly reduces the process rate (Figure 7b,e). Surface modification with 1 × 10^−2^ M solution does not provide any significant inhibition of metal corrosion either. A further rise in the concentration of the modifying solution to 0.1 M increased the inhibitory effect, and a reduction of the rate of corrosion was observed (Figure 7c, compare curves 1 and 3). The initial stages of dissolution were studied by piezoquartz nanoweighing (Figure 7c,d). The presence of a siloxane layer leads to corrosion inhibition in the first 2 h. Even deposition from solution with a concentration equal to 1 × 10^−4^ M provides a more than two-fold decrease in the rate of corrosion process, and a rise in the solution concentration to 0.1 M brings more than a five-fold decrease. Resistometry (Figure 7) and gravimetry after solution corrosion tests (Figure 9) confirm these results. Thus, relatively “thick” surface layers obtained by modifying the surface with 0.1 M VS solution efficiently inhibit the metal corrosion (i.e., provide more than a two-fold reduction in corrosion losses of the metal).

The stability of the surface organosilicon film was estimated by means of an IR spectroscopy: Reflective IR spectra of the zinc surface modified with vinylsilane solutions were recorded after three days of testing in a sodium chloride solution.

**Figure 10:**
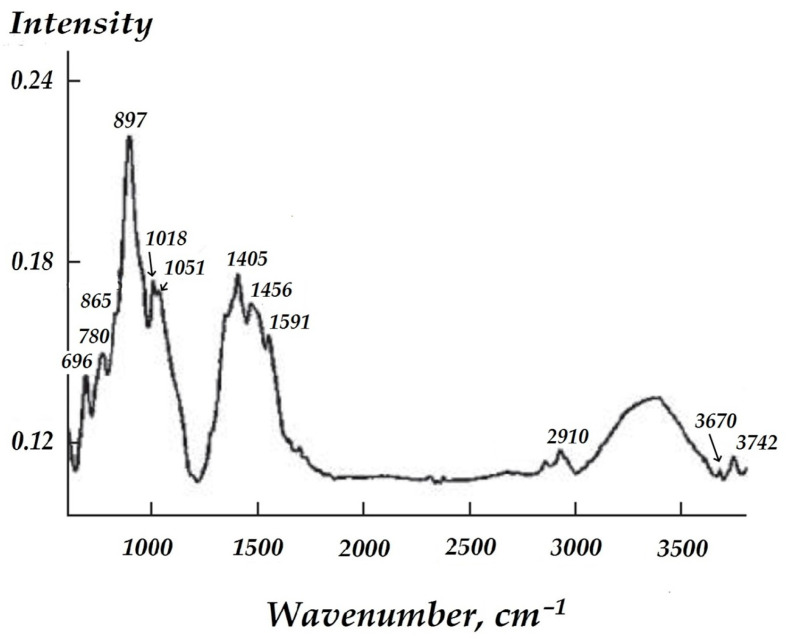
FT-IR spectra of the surface of sputtered zinc with vinylsiloxane layer. Three days of testing in 0.1 M NaCl.

From Figure 10 can see that FT-IR spectrum exhibits bands located near: 846, 897, 966, 1013, 1038, 1404, 1462, 1551, 2859, 2930, 3673, 3744 cm^−1^.

## 4. Discussion

An analysis of the vinylsilane adsorption isotherm on freshly deposited zinc shows that it (Figure 1) corresponds to the polymolecular adsorption isotherm. At low solution concentrations (<1 × 10^−4^ M), a surface coverage of less than a monolayer was observed. Judging by the Langmuir isotherm [46], a monolayer on the surface is achieved at a concentration of 1 × 10^–4^ M of the VS solution. Figure 1 shows that at such a concentration of VS, about 20 ng of vinyltrimethoxysilane is adsorbed on the zinc, which corresponds to 3 mol/nm^2^ of the surface. Calculation of the molecule geometry showed that, in the case of dense monolayer packing, 2.4 molecules of vinyltrimethoxysilane can be arranged per nm^2^ of the surface, while in the case of silane hydrolysis in H_2_O [47], 4.34 molecules per nm^2^ of vinilsilanol can be found, which corresponds to a thickness of about 0.9 nm.

To understand the mechanism of the formation of surface layers, experimental data on the adsorption of vinyltrimethoxysilane from an aqueous solution were processed using well-known adsorption approaches. The experimental data are correlated with adsorption approximations: Langmuir, BET [48], Temkin [49], Flory–Hagtins [50], Langmuir–Freindlich [51], and the multicenter Langmuir isotherm [46,51].

Langmuir isotherm (6):

(6) $$1/C=1/m_{m}+C(1/m_{m}b)$$

where *C* is the concentration of VS (M); *m_m_* is the monolayer capacity in the case of dense coverage (ng); *b* is constant;

BET isotherm (7):

(7) $$C/m(1-C)=1/m_{m}k+C(k-1)m_{m}k$$

where *m* is the mass of adsorbed VS (ng); *k* is constant.;

Flory–Huggins isotherm (8):

(8) $$\ln(\theta /c)=x-1+\ln K+x\ln{\left(1-\theta \right)}^{x}$$

where *θ* is the degree of surface coverage; *x* is the substitution factor, i.e., the number of water molecules displaced from the surface during the VS adsorption; and *K* is the adsorption equilibrium constant;

Temkin isotherm (9):

(9) $$\theta =a+\frac{1}{f}\ln c$$

where *a* is constant that can be used to calculate the adsorption equilibrium constant; *f*—the factor of surface heterogeneity;

Langmuir–Freundlich isotherm (10):

(10) $$\log\left[\frac{\theta }{(1-\theta }\right]=h\log K+h\log c$$

where *h* is the degree of heterogeneity of the surface;

Langmuir multicenter isotherm (11)

(11) $$\log(\theta /c)=\log K+n\log{\left(1-\theta \right)}^{}$$

where *n* is the number of adsorption centers that can be occupied by an adsorbate molecule.

The heat of adsorption ∆G^o^_ads_ has been determined from Equations (6)–(11) after the definition of the adsorption equilibrium constant K from Equation (12) [51,52]:

(12) $$K=\frac{1}{55.5}\exp\left(\frac{-\Delta G_{ads}^{0}}{RT}\right)$$

Correspondence of the VS adsorption isotherm on zinc to the generally accepted adsorption approximations (namely, correlation coefficients) and the adsorption characteristics of HS molecules, including the values of adsorption heats, are presented in [53].

Representation of the isotherm of adsorption of VS in terms of Langmuir (6) and BET (7) equations (in the coordinates of the linear form of the equations) made it possible to determine the monolayer capacity, which was 23.42 and 42.13 ng when calculating using the Langmuir equation and the BET equation, accordingly. The recalculation of the monolayer capacity into coverage of the surface by the molecules of adsorbate indicated that from 2.4 to 4.3 molecules are adsorbed on the 1 nm^2^ of the surface in the case of monolayer coverage, and the area occupied by one VS molecule ranges from 0.455 to 0.231 nm^2^ respectively. An estimation of the conditional radii of -Si-O-C-H and -Si-O-H fragments based on reference values of lengths of chemical bonds and radius of atoms showed that the total length of the methoxy fragment is 0.725 nm, and that of the silanol fragment is 0.541 nm. These values correspond to the next magnitudes of “landing sites” of an individual molecule amounting to 0.413 and 0.229 nm^2^/molecule, which is equivalent to the coverage of 1 nm^2^ of zinc surface with 2.42 vinylsilane molecules or 4.35 vinylsilanol molecules. Satisfactory agreement between theoretically calculated and experimentally obtained values of surface coverage suggests that the molecules are arranged vertically on the metal surface. In addition, processing of adsorption data using the approaches applied to describe adsorption in solutions (Equations (6)–(11)) showed that the adsorption of VS on zinc is of “substitution” type since the adsorption isotherm is reliably outlined by the equation of Flory–Huggins (8). Nearly always, there is adsorbed water (H_2_O_ads_) on the surface under natural conditions. The H_2_O_ads_ is displaced from the surface in the course of adsorption. Processing according to Equation (8) made it possible to find the numerical value of x to be 6.9. This implies that during adsorption, one molecule of VS displaces more than six H_2_O_ads_ molecules from the surface of zinc. Moreover, one molecule of vinylsilane lands on the zinc surface by occupying more than one adsorption site on it. To determine the number of adsorption centers on the surface per 1 VS molecule, for adsorption data processing, the Langmuir multicenter adsorption isotherm (11) was applied, the basic assumption of which includes the presence of the surface of adsorption centers. The plot of adsorption results in the Equation (11) coordinates is a straight line with a correlation coefficient above 0.9 [53]. This indicates the applicability of the Langmuir multicenter adsorption isotherm to the adsorption of VS on zinc. The value of n was 6.9, i.e., each adsorbate molecule of vinylsilane occupies 6.9 centers on average, which indicates that 1 nm^2^ accounts for 15 to 30 adsorption centers. These data have been reaffirmed by the IR results. The spectrum is shown in Figure 3, and the assignment of bands in the spectrum can be found in [53]. In the spectrum (Figure 3), a broad intense band at about 3400 cm^−1^ can be seen, corresponding to the surface hydroxyl groups that remain “free” after vinylsilane adsorption, i.e., vinylsilanol interacts only with a part of hydroxyl groups without affecting a major portion of them. Figure 3 shows that FT-IR spectra of surface VS nanolayers with various thicknesses have the same shape (Figure 3, curves 1–4), which indicates the similarity of the chemical composition of the layers obtained. The spectrum (Figure 3) analysis shows that the bands corresponding stretching vibrations of -OH radicals (near 3450–3500 cm^−1^) and bending vibrations of H-O-H (near 1591, 1550 cm^−1^) [51] are exhibited most intensely. That indicates, as mentioned above, the presence of hydroxyl groups on the zinc surface. The IR spectrum of a zinc surface covered by a layer of VS (Figure 3) contains bands corresponding to the vinylsiloxane layer generated after silane molecule hydrolysis and their subsequent condensation (and eventually polycondensation) on the surface. For example, IR-bands near 780 and 826 cm^−1^ and an intense broad band located in the 1080–1090 cm^−1^ region satisfied to Si-O-Si fragment vibrations and the bands at 1185 and 1203 cm^−1^ can be ascribed to vibrations (symmetric and asymmetric of the bonds of Si-O in Si-O-Si [53,54,55,56]. The band around 1092 cm^−1^ can also be attributed to vibrations of the Si-O-Si fragment [54] and the band at 2910 cm^−1^ to the stretching vibrations of the C-H bond [55].

Obtained data indicate a surface polycondensed siloxane layer formation. Such a layer is formed already at VS solution concentration equal to 1 × 10^−6^ M. An increase in the VS solution concentration from 1 × 10^−6^ to 1 × 10^−4^ M does not enhance the intensity of the IR-bands corresponding to Si-O-Si (Figure 3, curves 1 and 2), and the intensity of these bands even decreases. An increase in the intensity of these bands was observed only with a rise in the concentration of the solution up to 0.1 M (Figure 3, curve 4), in which, apparently, a polycondensation reaction proceeds more completely. Spectrum (Figure 3) also contains bands attributed to the functional group of the molecule of the silane. For example, 1590 cm^−1^ band lies in the region near the area of vibrations of the double bond -CH=CH2, and the bands located near 2909 and 2842 cm^−1^ can be attributed to the -C-H bond stretching vibrations. 1456 cm^−1^ band can be assigned to the C-H bonds’ deformation vibrations [57]. Bands near 763, 865, and 1270 cm^−1^ attribute to Si-C bonds’ vibrations.

There are bands at 679, 696, 966, and 3740 cm^−1^ in all spectra. These bands can be attributed to the vibrations of the Si-OH silanol group [55,58]. The 3670 cm^−1^ band may correspond to the hydrogen bond vibrations. Moreover, the spectrum of a metal surface covered by layers that formed after surface treatment by solutions with low VS concentrations (Figure 3, curves 1 and 2), a band at about 611 cm^−1^ was observed (Figure 3, curve 4), which was also observed after surface pretreatment by 0.1 M VS solution (Figure 3, curve 4). In all the spectra obtained (Figure 3), low-intensity bands were found in the interval of 890–920 cm^−1^, which corresponds to the vibrations of surface -Me-O-Si metalolsiloxane groups (where Me is a metal atom, in this case Zn) [51,55,56]. Thus, the analysis of the spectra showed that, on the freshly sputtered zinc surface, vinyltrimethoxysilane is hydrolyzed according to reaction (13) with the formation of silanol, while in the case of layers generated in solutions with low concentrations, the hydrolysis reaction does not go to completion, and some of the molecules contain methoxy (-OCH_3_), not silanol (-OH) groups. Apparently, these molecules do not undergo condensation and polycondensation reactions. At higher concentrations of the VS solution, all silane molecules are completely hydrolyzed and enter into a polycondensation reaction to give siloxane oligomers on the surface. In this case, the silanol molecules react (enter into condensation) with the metal surface hydroxyl groups with the formation of stable zinc-siloxane (Zn-O-Si) bonds (Equation (14))

(13) $$CH_{2}=HCSi{\left(OCH_{3}\right)}_{3}+H_{2}O\to CH_{2}=CHSi{\left(OH\right)}_{3}$$

(14) $$CH_{2}=CHSi{\left(OH\right)}_{3}+HO-Zn-\to CH_{2}=CH{\left(OH\right)}_{2}Si-O-Zn-$$

Our research has allowed us to propose a mechanism for the formation of surface layers. A sequential scheme illustrating the formation mechanism of a vinyl-containing siloxane layer on a zinc surface is shown in [53]. Investigations of zinc surface after vinilsilane adsorption by means of FT-IR spectroscopy had shown that both vinylsilanol and vinylsilane (non-hydrolyzed) molecules are present on the surface at pre-monolayer coverage. Vinylsilanol molecules adsorbed under conditions of pre-monolayer (less than one monolayer) surface coverage react with each other and with hydroxyl groups on the surface to form siloxane fragments firmly bound to hydroxide groups of metal surface [53]. The processing of adsorption data in terms of the Temkin isotherm approach (Equation (9)) confirms the strong bond formation between VS and the surface since the value of homogeneity degree from reaction (9)) equals 17.65 [53], which is close in order of magnitude to the values determined from isotherms of anion-specific adsorption on the sleek platinum surface [59].

Thus, the adsorption data analysis show that, at the initial stages, VS adsorption occurs with the displacement of H_2_O_ads_, where each silane or silanol molecule occupies more than six adsorption pads on the zinc surface, and these molecules are arranged vertically. Closely spaced (neighboring) molecules react with one other, resulting in the formation of siloxane low molecular weight oligomer compounds—most likely dimers or trimers, which are bound to the metal surface either by hydrogen bonding or by chemical covalent links. The concentration of the VS solution’s increase to 0.1 M causes a more complete hydrolysis of vinylsilane molecules at the surface and the formation of a nanosized siloxane oligomeric film with an ordered structure on the zinc surface. The value of the degree of oligomerization was defined from the IR spectra after exposure for 10 min in the VS solution by determining the ratio of the intensity values of the bands related to the corresponding compounds. The value of the oligomerization degree was n = 8–12. The oligomeric chains on the surface link to one another by hydrogen bonding [53] can be seen from the corresponding band in the IR spectrum (Figure 3). The oligomeric film’s total thickness is 10–12 nm or 10 molecular layers.

**Figure 11:**
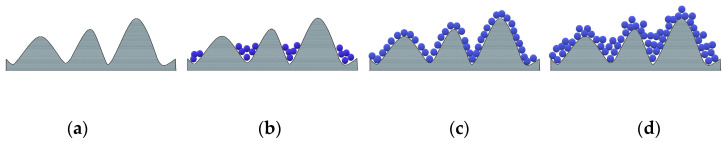
Schematic representation of VS adsorption in an aqueous solution on freshly sputtered zinc. Compiled based on the AFM results. (**a**) initial surface of zinc; the zinc surface after VS adsorption: (**b**) pre-monolayer coverage; (**c**) one VS monolayer; (**d**) polymolecular coverage.

Atomic force microscopy (AFM) was also used for studying the adsorption of vinyltriethoxysilane from solution on the freshly sputtered zinc surface. The AFM study results are presented in Figure 4 and Table 1. It can be seen from Figure 1 that with pre-monolayer coverage of the surface of zinc by VS molecules, the average surface roughness decreases. This, apparently, is because the filling of pore cavities by molecules of adsorbed vinylsilane somewhat reduces the total porosity (Figure 11b) of an initial metal surface layer (shown in Figure 11a), and hence, the average surface roughness (Table 1).

Therefore, the vinylsilane adsorption causes variations in the values of the metal surface average roughness (Table 1). For example, if the surface is covered with one monolayer of the silane, the roughness somewhat increases compared to pre-monolayer coverage (Table 1), apparently because the uniform distribution of the adsorbed layer over the surface, and the adsorbate film repeats the surface geometry, including in pores (Figure 11c). Further (polymolecular) adsorption causes decreasing porosity compared to the pre-monolayer and monolayer coverages (Figure 11d) due to the filling of pores and, as a result, a decrease in the average roughness value (Table 1).

The influence of siloxane surface layers on the Zn electrochemical ongoing has been investigated. It has been established that the corrosion potential of the metal after metal modification in a vinylsilane solution shifts to the positive side (Figure 5, curves 2–5), which may indirectly indicate that the surface siloxane layers can inhibit the anodic process of metal dissolution [60,61]. As can be seen from Figure 6b, the presence of a siloxane nanolayer on the zinc surface slows down the process of anodic dissolution. Thus, one monolayer of VS causes a decrease in the anodic current by almost a factor of two (see, for example, Figure 6b, compare curves 1 and 2; Table 3). Increasing the film thickness to 5–7 molecular layers (by deposition of VS from 0.01 M solution) causes a more than three-fold reduction in the current of anodic dissolution of Zn and increased the thickness to 10 molecular layers, providing its almost four-fold reduction (Figure 6b, curve 4; Table 3). However, a further rise in the layer thickness did not provide the corresponding reduction in the anodic current (Figure 6b, curve 3, Table 3).

It was also shown (Table 2) that the presence of a vinylsilane layer on the surface shifts the metal pitting potential to more positive values, which, according to [36], may indicate the inhibition of local anodic dissolution of zinc. Visual observation of the external state of the zinc surface showed that in the absence of a surface siloxane layer at a potential of –0.75 V, intense anodic pitting zinc dissolution occurs, and the pitting depth reaches 1 mm (Figure 6a). On metal samples with a single siloxane monolayer, pitting nucleation centers are seen (Figure 6c, left image). An increase in the film thickness to several molecular layers leads to uniform dissolution of the metal (Figure 6c, right image) since films of this thickness are apparently ordered and most efficiently prevent the penetration of anodically active electrolyte particles to the surface and metal dissolution.

Thus, it has been found that surface siloxane films having thickness of 2–3 or more molecular layers are most efficient for inhibiting the local zinc anodic dissolution. Apparently, films of this thickness have a well-ordered structure, and a further increase in the thickness of the surface siloxane film does not provide a proper further increase in anti-corrosion action.

The study of the corrosion properties of zinc demonstrated that self-assembled surface siloxane layers are able to inhibit zinc corrosion. Figure 7 and Figure 8 show the results of accelerated corrosion tests of zinc carried out in a climatic chamber. Using image processing methods, evaluating the surface coverage with corrosion products (Figure 7b,d) and gravimetrically (Figure 7c,e), it was shown that siloxane surface films reduce the corrosion rate of zinc. The monolayer coating slightly inhibits corrosion. Polymolecular films provide a two-fold decrease in the corrosion rate during long-term testing for more than 3 months (Figure 7c–e). These results are confirmed by resistometric data (Figure 7f), which showed that the corrosion rate of freshly deposited zinc decreased more than threefold in the presence of 10 molecular layers of siloxane film. The corrosion rate under a monolayer coverage under the same conditions decreases only one and a half times (Figure 7f).

It has been established by the study of uniform corrosion kinetics that multilayer surface coverage is required to inhibit metal dissolution. For example, it has been found that a film with a thickness of one and two monolayers slightly reduces the rate of the process. An increase in thickness reduces the corrosion rate more than two-fold (Figure 7f). The initial dissolution stages were studied by piezoquartz nanobalance (Figure 7c), and it was shown that two siloxane layers reduce the corrosion rate. Even in the case of monolayer coverage, there is a more than two-fold decrease in the corrosion rate, and a rise in the thickness of 10 molecular layers leads to a more than five-fold decrease. These data were also confirmed in the tests in a chloride-containing solution with a gravimetric determination of corrosion performed using an analytical weighing (Figure 9).

An IR assessment of the state of the vinylsiloxane layer after corrosion tests in solution showed (Figure 10) that the surface layer was resistant to the corrosive electrolyte action. IR spectra taken after 72 h of testing in a chloride-containing solution (Figure 10) confirmed the presence of a siloxane layer on the zinc surface. A number of bands were found in the spectrum that corresponded to fragments of the siloxane layer and to the vinyl group. Moreover, an intense band at about 897 cm^−1^ corresponding to a metal-siloxane bond (Zn-O-Si) was observed in the spectrum. This may indicate that after corrosion testing, a siloxane layer firmly bonded to the surface is present. FT-IR-spectrum (Figure 10) showed bands corresponding to vinylsiloxane compounds.

In fact, the bands at 780, 826, and 1080–1090 cm^−1^ correspond to Si-O-Si vibrations, and the bands at 1185 and 1203 cm^−1^ can be correlated with Si-O-Si vibrations. The spectrum also contains bands corresponding to vibrations of the Si-C bond (1018 cm^−1^), double bond -CH=CH_2_ (1590 cm^−1^), stretching vibrations (2909, 2842 cm^−1^), and bending vibrations (1456 cm^−1^) of the C-H bond [51,52,53,54,55].

The data obtained confirm the presence of a polycondensed siloxane layer on the zinc surface. Thus, it was shown that the siloxane layers on the surface of zinc are preserved in an aqueous chloride-containing solution for at least 72 h, which indicates the presence of a strong interaction between the nanolayer and the surface, which, apparently, is determined by the formation of hydrolytically stable Zn-O-Si covalent bonds. The IR spectrum of the zinc surface after corrosion tests contains bands corresponding to the silanol groups (679, 696, 966, and 3742 cm^−1^), hydrogen bonds (3670 cm^−1^), methoxy groups of vinyltrimethoxysilane (611 cm^−1^), and Me-O-Si groups (890–920 cm^−1^) [54,55,56].

## 5. Conclusions

1. It has been established that vinylsilane adsorbed on the surface of thermally deposited zinc from solution displaces adsorbed water from the surface, occupying more than six adsorption pads.
2. It has been found that vinylsilane adsorbed on the surface of thermally deposited zinc from solution displaces adsorbed water from the surface, occupying more than six adsorption pads.
3. It has been defined that vinylsilane adsorbed on the surface of thermally deposited zinc from solution displaces adsorbed water from the surface, occupying more than six adsorption pads.
4. A VS solution concentration increase to 0.1 M leads to the creation of polycondensed siloxane oligomeric film with a degree of polycondensation *n* = 8–12 on the surface. The surface oligomeric fragments are linked to one another through hydrogen bonds and to the surface through Zn-O-Si bridge bonds. The total film thickness is 10–12 nm, or 10 molecular layers.
5. The resulting siloxane layers inhibit the zinc anodic dissolution of and zinc corrosion in chloride-containing electrolytes and in the atmosphere conditions, and it can also reduce the rate of metal pitting dissolution.
6. It has been found that a monomolecular siloxane layer is insufficient to suppress the zinc corrosion processes. Corrosion and localized dissolution of zinc are most efficiently inhibited by siloxane layers with a thickness of more than 2–3 molecular layers. At this thickness, the most ordered surface structures are apparently formed.
7. The self-organizing surface siloxane layers formed on zinc are stable to the action of sodium chloride solution and, though corrosion processes occur, they retain durable bonds with the metal surface.

## Figures and Tables

**Table 1:** Mean zinc surface roughness upon VS adsorption from an aqueous solution according to AFM data.

Concentration of the VS Solution	Mean Surface Roughness (AFM Data), nm
0 M VS	17.006
1 × 10^−6^ M VS	6.957
1 × 10^−4^ M VS	14.706
1 × 10^−1^ M VS	11.157

**Table 2:** Influence of pretreatment of the zinc surface by a vinylsilane solution on the metal pitting potential.

N	Systems	E_pit_, V (SHE)	ΔE, V
1	Zn	−0.934	0
2	Zn + 1 × 10^−4^ M VS	−0.84	0.094
3	Zn + 1 × 10^−2^ M VS	−0.793	0.141
4	Zn + 1 × 10^−1^ M VS	−0.784	0.15

**Table 3:** Effect of the preliminary modification of zinc with vinylsilane on the anodic dissolution of zinc.

Systems	Unmodified Zn-	Zn Modified by 0.0001 M VS	Zn Modified by 0.001 M VS	Zn Modified by 0.01 M VS	Zn Modified by 0.1 M VS	Zn Modified by 0.3 M VS	Zn Modified by 0.5 M VS
Anodic current density ia, mA/cm^2^	0.76	0.42	0.31	0.22	0.19	0.24	0.31
Inhibition coefficientг	-	1.82	2.46	3.47	3.95	3.18	1.36

## Data Availability

Not applicable.
